# MBGD update 2018: microbial genome database based on hierarchical orthology relations covering closely related and distantly related comparisons

**DOI:** 10.1093/nar/gky1054

**Published:** 2018-11-20

**Authors:** Ikuo Uchiyama, Motohiro Mihara, Hiroyo Nishide, Hirokazu Chiba, Masaki Kato

**Affiliations:** 1 Laboratory of Genome Informatics, National Institute for Basic Biology, National Institutes of Natural Sciences, Nishigonaka 38, Myodaiji, Okazaki, Aichi 444-8585, Japan; 2 Data Integration and Analysis Facility, National Institute for Basic Biology, National Institutes of Natural Sciences, Nishigonaka 38, Myodaiji, Okazaki, Aichi 444-8585, Japan; 3 Dynacom Co., Ltd. 5-1-27, Onoedori, Chuo-ku, Kobe, Hyogo 651-0088, Japan; 4 Database Center for Life Science, Research Organization of Information and Systems 178-4-4 Wakashiba, Kashiwa, Chiba 277-0871, Japan

## Abstract

The Microbial Genome Database for Comparative Analysis (MBGD) is a database for comparative genomics based on comprehensive orthology analysis of bacteria, archaea and unicellular eukaryotes. MBGD now contains 6318 genomes. To utilize the database for both closely related and distantly related genomes, MBGD previously provided two types of ortholog tables: the standard ortholog table containing one representative genome from each genus covering the entire taxonomic range and the taxon specific ortholog tables for each taxon. However, this approach has a drawback in that the standard ortholog table contains only genes that are conserved in the representative genomes. To address this problem, we developed a stepwise procedure to construct ortholog tables hierarchically in a bottom-up manner. By using this approach, the new standard ortholog table now covers the entire gene repertoire stored in MBGD. In addition, we have enhanced several functionalities, including rapid and flexible keyword searching, profile-based sequence searching for orthology assignment to a user query sequence, and displaying a phylogenetic tree of each taxon based on the concatenated core gene sequences. For integrative database searching, the core data in MBGD are represented in Resource Description Framework (RDF) and a SPARQL interface is provided to search them. MBGD is available at http://mbgd.genome.ad.jp/.

## INTRODUCTION

The microbial genome database is expanding rapidly due to advances in sequencing technology, revealing the great diversity of the microbial world from two directions. On the one hand, our knowledge about the entirety of microbial diversity is still expanding through genome or metagenome sequencing of samples extracted from various environments. On the other hand, comparison among the genomes of the same species revealed a large diversity of species genomes, which is often represented as a pan-genome (1), i.e. the entire gene repertoire of a given species.

We have been developing the Microbial Genome Database for Comparative Analysis (MBGD), which provides orthologous relationships among microbial genomes published so far as a basis for comparative analysis of either closely related or distantly related genomes (2,3). For this purpose, MBGD originally maintained all-against-all similarities among all the translated sequences of the stored genomes, and allowed a user to create an ortholog table (a set of ortholog groups) from any specified set of genomes, in addition to the precomputed ‘default’ ortholog table (2,4). More recently, to reduce the computational burden for dynamic calculations, MBGD provided two types of precomputed ortholog tables: the standard (default) ortholog table containing one representative genome from each genus covering the entire taxonomic range, and taxon specific ortholog tables containing the genomes belonging to each taxonomic group (3,5).

The problem with this approach is twofold. First, rapid accumulation of the genomic data of the same or closely related species expands the size of all-against-all similarity data substantially, while the increased net amount of information (i.e., the size of gene repertoire) is limited. Second, the standard ortholog table contains only genes that are contained in the representative genomes, and thus a considerable amount of information may be lost from the standard ortholog table, considering within-species and within-genus genomic diversity. To address these problems, we developed a stepwise protocol to construct ortholog tables in a bottom-up manner, i.e. from within-species ortholog tables to within-genus ortholog tables to the standard (between-genus) ortholog table covering the entire taxonomic range.

Here, we introduce the recent development of MBGD including the above fundamental modifications to the data construction strategy, as well as several new functionalities that enhance the usability of MBGD.

## DATA SOURCES

MBGD incorporates all complete genome sequences of bacteria, archaea, and unicellular eukaryotes including fungi and protozoa available at the NCBI genomes FTP site. We referred to the information in the ASSEMBLY_REPORTS directory and incorporated the RefSeq entries (6) whose assembly levels were ‘Complete genome’ or ‘Chromosome’. We also incorporated the original GenBank entries without the corresponding RefSeq entries whose assembly levels were ‘Complete genome’ (for both prokaryotes and eukaryotes) or ‘Chromosome’ (for eukaryotes only). We further checked the quality and completeness of all data and retained only the data that satisfied the following conditions: (i) The ratio of the gap characters (‘n’) in the sequence was <1% (for prokaryotes) or <20% (for eukaryotes); (ii) The number of CDS was ≥100 and the number of CDS per genome length (kb) was ≥0.5 (for prokaryotes) or the number of CDS was ≥200 (for eukaryotes); (iii) the ratio of the length of unlocalized scaffold was <5% (for prokaryotes) or <25% (for eukaryotes). As a result, 1576 new genomes were added to the database, bringing the total number of genomes to 6318, including 5861 Bacteria, 254 Archaea and 203 Eukaryota. The number of unique species and genera are 2547 and 1019, respectively.

## BOTTOM-UP CONSTRUCTION OF HIERARCHICAL ORTHOLOGY RELATIONSHIPS

Previously, MBGD calculated all-against-all similarities among the stored genomes and created the standard ortholog table and a taxon specific ortholog table for each taxon in each taxonomic rank independently using a hierarchical ortholog clustering program, DomClust (7). For creating the standard ortholog table, the clustering results were further refined using the DomRefine program (8) based on multiple sequence alignment and phylogenetic tree construction.

**Figure 1.**
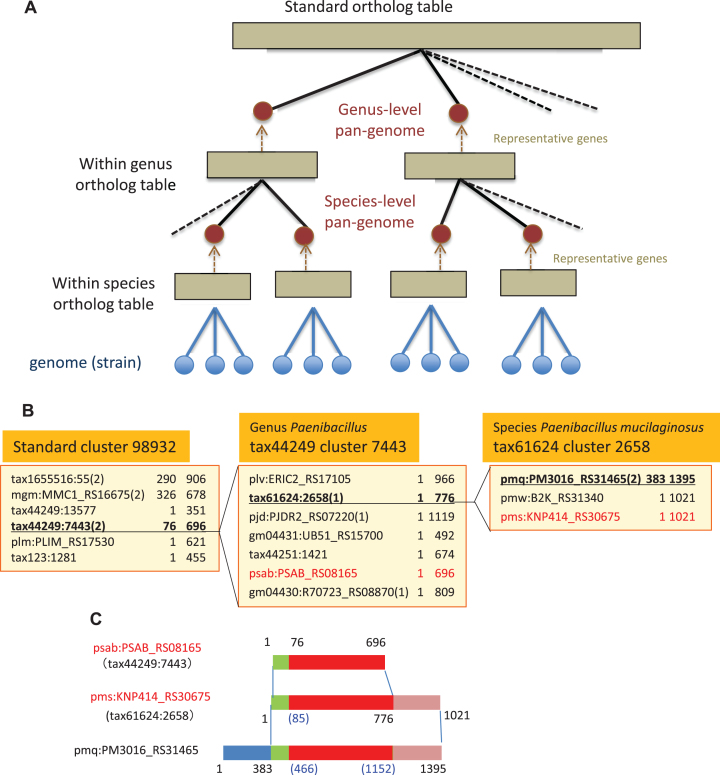
The bottom-up procedure for constructing hierarchical orthology relationships. (**A**) Overview of the procedure. The procedure progresses from bottom to top. (**B**) Hierarchical ortholog groups. Here, the construction process goes from right to left and the expansion process goes from left to right. A representative gene in each cluster is indicated in red, and the target clusters to be expanded are underlined. A gene in a pan-genome is represented as ‘taxid:clustid’, which is actually the representative gene of the cluster. The number in parentheses is the domain number and the two numbers after each gene name are the beginning and end positions of the domain. (**C**) Domain boundary mapping between clusters at different levels. The example is the same as in B. The red segment corresponds to the domain tax44249:7443(2) in the standard cluster 98932. Missing positions by this mapping are filled by a simple linear interpolation, shown by the numbers in parentheses.

The new protocol constructs these ortholog tables from lower to higher taxonomic ranks in a stepwise manner (Figure 1). First, for each species having at least two genomes, all-against-all similarities among the genomes belonging to that species are calculated and a within-species ortholog table is created using DomClust; the species-level pan-genome is then created by picking one representative gene from each orthologous group. Next, for each genus having at least two species, all-against-all similarities among the species-level pan-genomes created in the previous step and other genomes belonging to that genus are calculated and a within-genus ortholog table is created; then the genus-level pan-genome is created by picking one representative gene from each orthologous group. Finally, all-against-all similarities among the genus-level pan-genomes are calculated and the standard ortholog table covering the entire taxonomic range is created.

To calculate within-species or within-genus all-against-all similarities, we used a faster but less sensitive similarity search program, UBLAST (9), while we used BLASTP (10) followed by Smith–Waterman alignment (11) as previously described (4) to calculate between-genera similarities. In this way, we can reduce the computation time required for all-against-all similarities.

During this calculation, a pan-genome is named with the taxonomy ID (taxid) of the species/genus and the representative gene is named with the cluster ID (clustid) of the ortholog group; thus each gene in a pan-genome is represented as taxid:clustid (Figure 1B). After the top-level clustering has been done, a gene in a pan-genome can be expanded with the members of the lower-level clusters (Figure 1B). Since DomClust performs a domain level clustering (7), the expansion process includes determination of the domain boundaries in each sequence (Figure 1C).

It is important how well the representative gene represents the ortholog group not only in terms of sequence similarity but also domain architecture. Here, we consider the following conditions for the representative gene selection: (i) the gene's length is close to the median length for the group, (ii) not a fusion gene, i.e. does not contain additional domains classified in other groups, (iii) not a fission gene, i.e. not a split gene of some gene in the same group, (iv) not an outlier, i.e. not dissimilar to the other member genes. Note that conditions (ii) and (iii) can be detected during the domain-aware classification of DomClust and condition (iv) can also be determined using the hierarchical clustering tree created by DomClust.

As a result of the pan-genome-based approach, the number of sequences for creating the standard ortholog table is 1.24 times larger than the previous representative genome-based approach, while these sequences can cover a total number of sequences that is almost 5 times larger (Table 1). On the other hand, the number of resulting clusters (including singleton) increased 1.56 times (Table 1), indicating that this approach tends to generate more small clusters.

**Table 1.** Comparison of data sizes between the current and the previous approaches

	Number of sequences ^a^	Number of clusters ^b^
Previous method (representative-genome-based)	3 735 085	491 920
New method (pan-genome-based)	4 640 598	768 073
Total sequences	22 521 946	

^a^The number of sequences used for creating the standard ortholog table.

^b^The number of clusters in the standard ortholog table.

## OVERALL DATA CONSTRUCTION PROCEDURE

**Figure 2.**
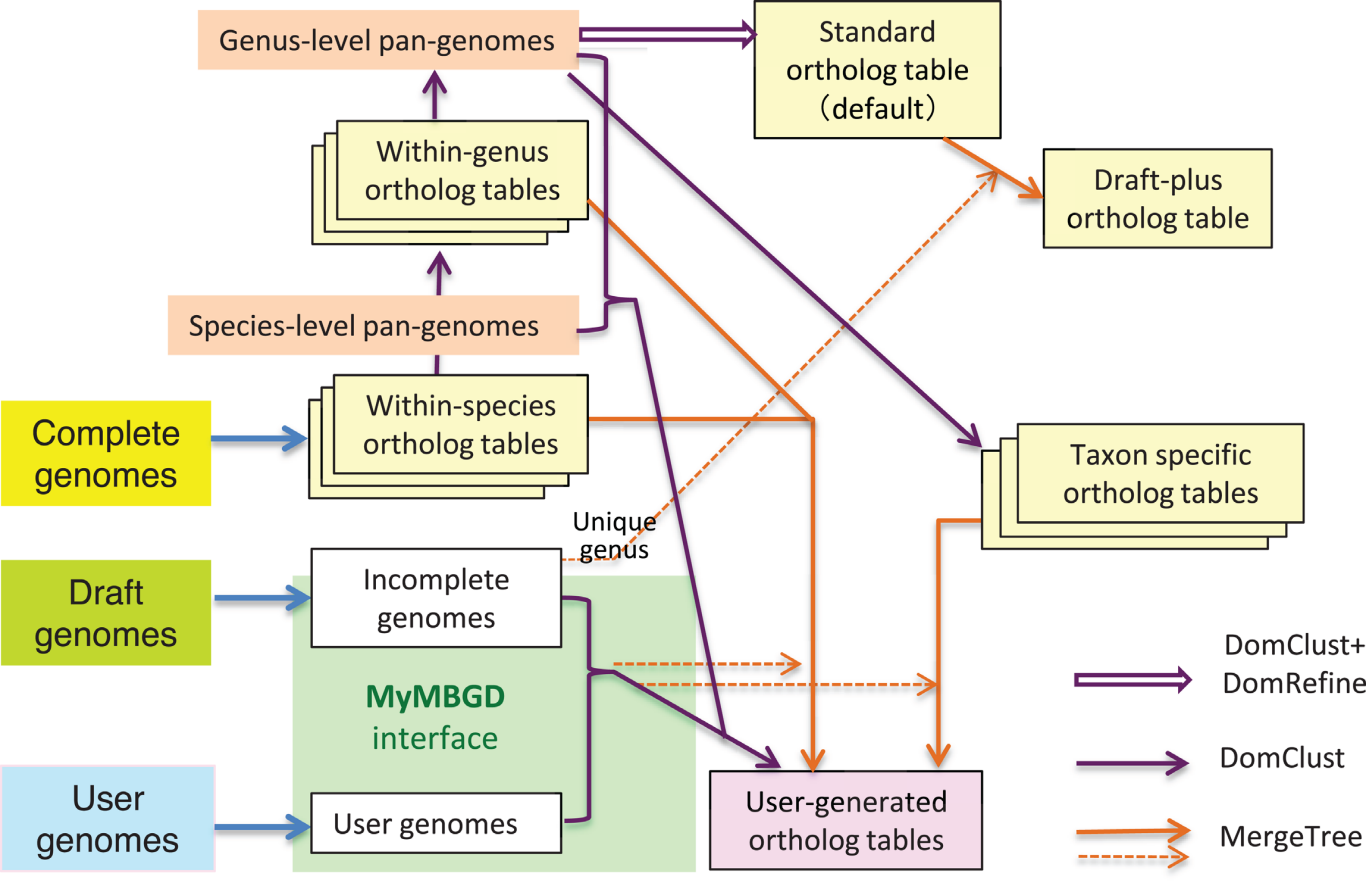
Overall procedure for constructing MBGD. This figure is an update of the previous version (3).

Reflecting the bottom-up procedure for orthology data construction introduced in the previous section, the overall data construction procedure has now been modified as shown in Figure 2. The standard ortholog table created from the genus-level pan-genomes using DomClust is further refined using the DomRefine program (8). Some draft genome sequences belonging to genera that are not included in the standard ortholog table are added to the standard ortholog table incrementally using the MergeTree program, generating the ‘draft-plus’ ortholog table (3). For each taxon at family level or above, we also created a taxon-specific ortholog table using DomClust with the genus-level pan-genomes belonging to that taxon as inputs, while taxon-specific ortholog tables at species or genus level have already been created during the bottom-up ortholog data construction procedure. Overall, most of the functionalities in the previous version are also provided in this version of MBGD.

## AN EXAMPLE OF A HIERARCHICAL ORTHOLOG GROUP

**Figure 3.**
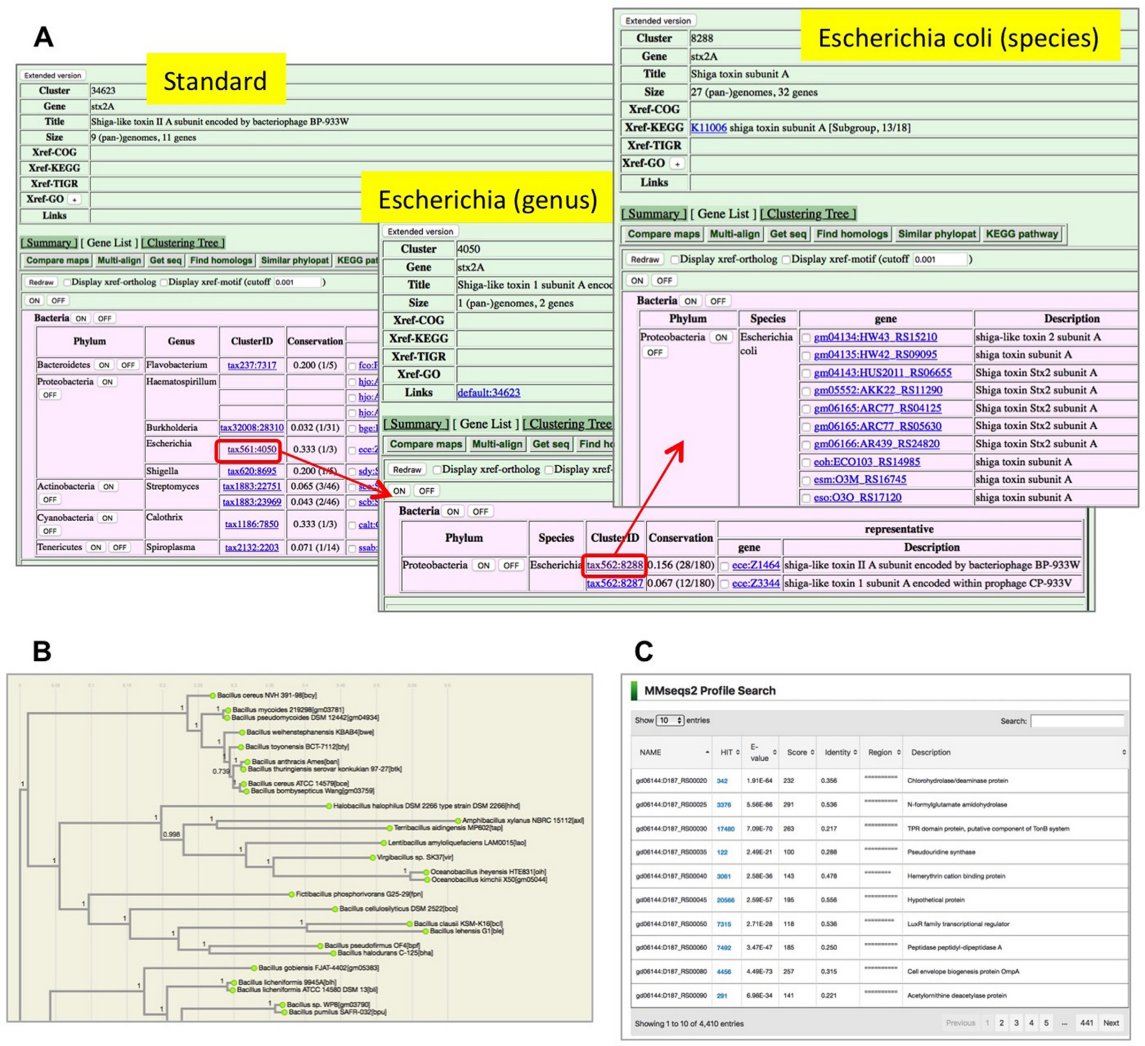
Screenshots of the new functionalities in MBGD. (**A**) An example of a hierarchical ortholog group. Shown is the ortholog group containing Shiga-like toxin subunit A. (**B**) A phylogenetic tree shown in the ortholog table summary viewer. Shown is a part of the phylogenetic tree created from the conserved orthologs of the family *Bacillaceae*. (**C**) The output of the profile search using MMseqs2.

As an example of a hierarchical ortholog group, here an ortholog group containing Shiga toxins (cluster ID 34623) is shown (Figure 3A). Shiga toxin (or Shiga-like toxin) is a well-known toxin produced by *Shigella dysenteriae* and some pathogenic strains of *Escherichia coli*, including O157. The top-level cluster contains 11 genes included in 8 genera pan-genomes (Figure 3A, left). Here, the ‘Conservation’ column shows the ratio of the species having this gene in each genus, and in this case only one or two species contain this gene in each genus, indicating a very sporadic distribution. Clicking the name in the ‘ClusterID’ column will take a user to the genus-level cluster page (Figure 3A, middle). Here, one can see that there are two paralogous lineages corresponding to type I and type II Shiga toxins, and the conservation ratios are quite different between them, suggesting some difference in their distribution patterns among *E. coli* strains. The distribution of each type among strains can then be examined by clicking the ‘ClusterID’ column again (Figure 3A, right). Alternatively, a user can expand the top-level cluster so as to include all members of the lower-level clusters by clicking the ‘Extended version’ button in the upper left corner.

Analysis of such sporadically distributed genes was often not possible in the previous version of MBGD because such an ortholog group was often not contained in the standard ortholog table, in which only one representative genome from each genus was incorporated. In fact, in this case, representative genomes of these genera were *E. coli* K12 and *Shigella flexneri* 301 whose genomes do not contain Shiga toxin.

## ORTHOLOG TABLE SUMMARY VIEWER

All available ortholog tables, either precomputed or user-defined, are listed in the ortholog table summary viewer (5), where a user can choose a taxon in the taxonomic tree shown in the left hand panel to see the selected taxon-specific ortholog table. Here a user can also switch the summary view of the current ortholog table shown in the right hand panel. Available views include a histogram of cluster size (Cluster size), a bar graph showing the relationship between occurrence pattern and functional category (Occurrence pattern), a similarity matrix of pairwise genome comparison (Pairwise comparison) and a diagram of syntenically conserved core structure created by the CoreAligner program (12) (CoreAlign). These functionalities are almost unchanged from the previous version.

For this version, we added another view, Phylogenetic tree, which shows the phylogenetic tree calculated using the concatenated alignment of the conserved core genes among the genomes in the ortholog table (Figure 3B). Here, multiple sequence alignment was created using Clustal Omega (13) and phylogenetic tree was calculated using FastTree (14). A phylogram is drawn using d3.phylogram.js (http://bl.ocks.org/kueda/1036776). For taxa at family level or below, the CoreAligner program was used to extract core orthologous groups taking account of synteny conservation, from which universally (100%) conserved ones in a one-to-one correspondence were used for phylogenetic tree calculation. For higher taxa, orthologous groups that were universally conserved in a one-to-one correspondence (without using CoreAligner) were used. For some very high level taxa (such as Bacteria) that had only very few such genes, we eliminated the organisms that had fewest genes conserved in ≥90% of the organisms until the number of core genes (conserved in ≥99% of the organisms) reached 50 or more.

## NEW INTERFACES FOR SEARCHING THE DATABASE

**Figure 4.**
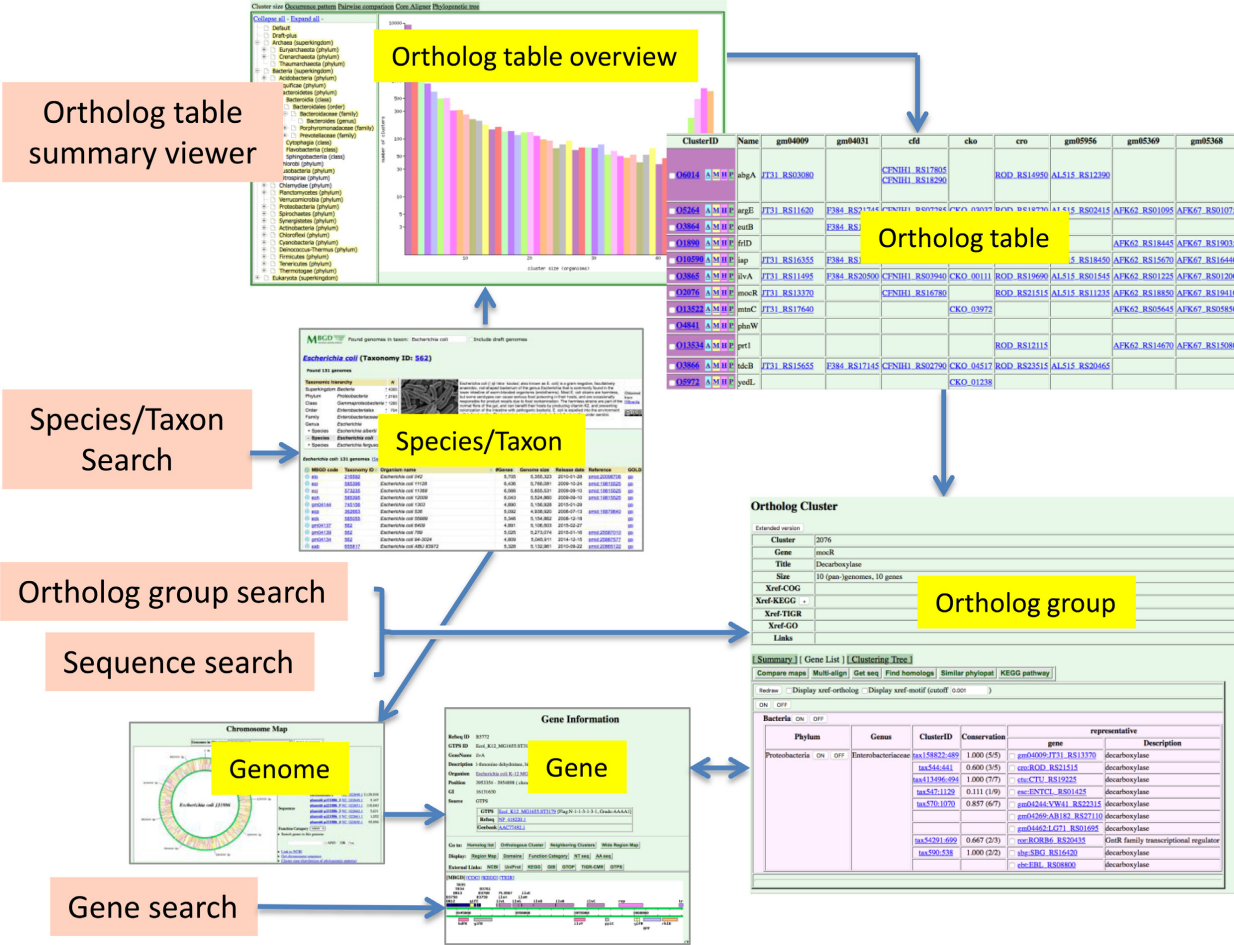
Interfaces for searching and browsing MBGD. Interfaces are shown in the light pink boxes.

The MBGD main page provides several interfaces for searching and browsing the database (Figure 4). These interfaces include: (i) a link to the ortholog table summary viewer, where a user can choose a taxon to see the taxon specific ortholog table; (ii) keyword search interfaces for searching against the ortholog table, gene entries, and organism/taxon names; and (iii) sequence search for user query sequences against the profile library constructed from the multiple sequence alignment of each ortholog group.

For keyword search against ortholog group or gene entries, the Elasticsearch engine (https://www.elastic.co/products/elasticsearch) is used to realize fast and flexible text search. For species/taxon search, the autocomplete functionality in the jQuery UI library (https://jqueryui.com) is used to help a user specify an appropriate species/taxon name. The result of the latter search is the species/taxon information page, which displays information about the specified species/taxon including the taxonomic position on the taxonomy tree, general information in the corresponding Wikipedia article obtained from the DBpedia project (https://wiki.dbpedia.org), and the list of available genomes belonging to that species/taxon.

For sequence search, we prepared profile libraries of ortholog groups for two programs: Hidden Markov Model profiles for the HMMER program (15) and the profile search version of the MMseqs2 program (16). Either program accepts multiple query sequences in FASTA format, and the results of the multiple queries are summarized as a list of the top hit ortholog groups for each query (Figure 3C). In particular, MMseqs2 is rapid enough to search the profile library even with a complete set of protein sequences in a bacterial genome as a query. Thus, this function can be used for the purpose of annotating a newly determined genome sequence.

## MBGD-RDF FOR INTEGRATIVE DATABASE SEARCHES USING SPARQL QUERIES

Comparative genomics based on orthology relationships is a key approach for integrating various aspects of biological knowledge, and utilizing Semantic Web technology, including Resource Description Framework (RDF) and the SPARQL query language for RDF, is a promising approach to integrate various resources distributed worldwide. We previously developed the Ortholog Ontology (OrthO) and converted the orthology data in MBGD into RDF (MBGD-RDF) using OrthO (17). Later, we developed Orthology Ontology (ORTH) (18) by integrating OrthO and another orthology ontology OGO (19), along with other existing ontologies used in the biological domains. Now, MBGD-RDF is re-created using ORTH.

We provide a simple interface for directly searching MBGD-RDF using a SPARQL query (http://mbgd.genome.ad.jp/sparql). Here, to help a user to write a SPARQL query, several example queries are provided in parameterized form, such as ‘search ortholog clusters by a specific GO term’. The result of a query is returned with the corresponding SPARQL code, which can be used as a template for making a more appropriate query.

MBGD-RDF has been used internally to implement some of the functions in MBGD, such as the species/taxon information page described above. Moreover, MBGD-RDF has been used in the collaborative development of an integrative microbial database under the MicrobeDB.jp project (http://microbedb.jp).

## DISCUSSION

The hierarchical orthology framework has previously been adopted for use in various methods and databases (20–25), and the idea of progressive orthology inference using taxonomic information in a bottom-up manner for improving efficiency has also been previously proposed (24,26). Here, we considered only taxa at species and genus levels as targets of hierarchical orthology inference, and comparisons among higher-level taxa were done independently. This is because substantial horizontal gene transfer events between distant lineages of bacteria and archaea may violate the validity of hierarchical orthology concepts. Nonetheless, the approach developed is effective for our purposes here, i.e. making an ortholog table that covers the entire set of genomes. Defining appropriate hierarchical relations among the higher-level ortholog groups remains an important future task.

The explosive increase in genomic data is ongoing and there is an increasing demand for further efficient platforms for handling and utilizing large-scale genomic data through comparative analysis. We have two problems to consider: one is to find a more sustainable strategy for updating the database, and the other is to facilitate effective use of our database for analyzing newly determined genomic and/or metagenomic data. For the former problem, we should consider some selection strategies for data incorporation. For the latter problem, the sequence search for a user's query provided in this release is the first step. We have a plan to develop more effective applications for analyzing a user's genomic data on the basis of orthology assignment using our database.
